# Diabetes and eating disorders: an exploration of ‘Diabulimia’

**DOI:** 10.1186/s40359-020-00468-4

**Published:** 2020-09-23

**Authors:** Sophie Elizabeth Coleman, Noreen Caswell

**Affiliations:** 1 Trainee Clinical Psychologist, University of Manchester, Manchester, UK; 2 Senior Lecturer, School of Psychology, University of Central Lancashire, Preston, UK

**Keywords:** Diabulimia, Diabetes, Type 1 diabetes mellitus, T1DM, Eating disorder, Insulin restriction, Insulin misuse

## Abstract

**Background:**

‘Diabulimia’ is the term given to the deliberate administration of insufficient insulin for the purpose of weight loss. Although Diabulimia can be life-threatening and prevalence rates in diabetes are high, there is a lack of research for how to effectively support people with the condition. This exploratory study aimed to provide much-needed information to healthcare professionals and guide the focus for future research.

**Methods:**

Forty-five individuals with Type 1 diabetes mellitus (T1DM) and a history of insulin misuse completed an online questionnaire. This included an assessment of their eating disorder psychopathology with the Eating Disorder Examination Questionnaire (EDE-Q) and 16 open-ended questions exploring their experience of Diabulimia. The responses to the open-ended questions were analysed using thematic analysis.

**Results:**

The average global EDE-Q score was 3.96 (1.21), which is consistent with eating disorder populations. Common themes identified were concerns about weight, difficulty coping with diabetes, past trauma, and the importance of relationships. Experiences with health professionals were overwhelmingly negative. Most participants had experienced serious medical intervention due to Diabulimia and were fully aware of the consequences of insulin restriction.

**Conclusions:**

Overall, individuals believed that a greater awareness of Diabulimia and more training for healthcare professionals is needed. While education on insulin misuse may be a necessary first step in treatment, psychological support is crucial. To deliver effective treatment, clinicians should be aware of the specific issues facing those with Diabulimia. The current study identified themes that clinicians may find useful to consider.

## Background

Diabulimia is an eating disorder experienced by people with Type 1 diabetes mellitus (T1DM). It is characterised by the deliberate restriction of insulin, resulting in weight loss [1]. Diabulimia is not currently acknowledged as a formal diagnosis [2], however in recent years it has gained attention in the press and been recognised within medical and psychiatric communities. For example, in 2017 there was a BBC documentary about the condition, titled *‘Diabulimia: The World’s Most Dangerous Eating Disorder’* [3]. In addition, in 2019 researchers at Kings College London were awarded £1.25 million to investigate Diabulimia over the next 5 years [4].

In 2017, the guidelines for the treatment of eating disorders by The National Institute for Health and Care Excellence (NICE) included a section on diabetes for the first time [5]. Although the term ‘Diabulimia’ was not recognised, guidance on how best to treat those who restrict insulin was included. The inclusion of these new guidelines demonstrates that those with T1DM and an eating disorder, specifically insulin omission, pose a unique problem [6].

### Eating disorders and T1DM

Within the literature there is a consensus that eating disorders are more prevalent in people with Type 1 diabetes mellitus (T1DM), especially females [7–11]. Eating disorders affect around 20% of females with diabetes and are twice as likely to occur in teenage girls with T1DM than in those without [12, 13]. Most commonly, eating disorders in people with diabetes take the form of disturbed eating behaviours (DEB) such as food restriction, binging and vomiting, laxative use and excessive exercise, which can occur with or without the presence of insulin misuse [9, 13]. There are aspects of diabetes management that may increase the risk of eating disorders, such as a higher body mass index (BMI) [9], concerns over shape and weight [14], difficulties coping with a long-term condition [13], and the effect of diabetes on self-image and family interaction [13]. Because of insulin treatment, adolescents with T1DM often see their weight increase substantially between their teenage years and early adulthood. This can exacerbate body dissatisfaction and lead to the development of DEB [12]. In addition, people with T1DM may feel overwhelmed by the constant focus on nutrition and medication [12]. The emergence of DEB may be an attempt to regain control over their eating and weight, thus leading to the development of an eating disorder.

### Insulin restriction and T1DM

As a result of insulin deficiency people often lose weight prior to their T1DM diagnosis, then rapidly gain weight once they begin insulin treatment [15]. Reducing or withholding insulin offers individuals with T1DM an easy way to achieve rapid weight loss. Blood glucose levels increase when insulin is withheld, resulting in a rapid breakdown of proteins (catabolism) as well as rapid calorie loss through glucosuria, an excretion of glucose in urine [16].

People who manipulate their insulin suffer severe consequences to their health. This includes diabetic ketoacidosis (DKA), a dangerous and acute complication of diabetes that occurs when the body has reduced insulin [13]. There are also serious long-term microvascular and macrovascular complications associated with Diabulimia, including retinopathy (vision loss) and nephropathy (kidney damage) [17–19]. Over the course of an eleven-year longitudinal study, it was found that insulin restriction at baseline increased the risk of death by 3.2 times [17].

Given the serious consequences of insulin restriction, it is incredibly worrying that the research suggests it is a common weight-loss behaviour among individuals with T1DM [11, 20–24]. One study found that an overwhelming 60% of those with T1DM self-reported insulin restriction [23].

### Predictors of insulin restriction

Several studies have reported that the fear of weight gain is a core component in the emergence of insulin mismanagement [10, 22, 25, 26]. Additionally, in one qualitative study participants who omitted insulin continued to do so due to rapid weight loss and the subsequent positive comments they received [10]. It was believed they had a *“*secret trick*”* to losing weight and felt in control of their bodies. Participants were often not aware of the long-term consequences of insulin omission until they experienced serious complications [10].

Although a common and life-threatening condition, there is a lack of research on Diabulimia, specifically on how best to prevent, detect and treat the condition. The omission of the label ‘Diabulimia’ in most research is likely because many researchers use formal diagnostic categories. However, individuals identify with the term ‘Diabulimia’ and may use it with healthcare professionals [6]. Therefore, it is important to investigate and understand the language used by those with lived experience.

### Aims of research

The aim of this study was to conduct an exploratory analysis into the views and experiences of people with lived experience of Diabulimia. This would provide healthcare professionals with an increased understanding of Diabulimia and pave the way for future research.

## Methods

### Design and participants

The research has a qualitative research design, using thematic analysis based on the principles described by Braun and Clarke [27]. Participants initially consisted of 55 individuals who stated they had T1DM and a history of insulin misuse for the purpose of weight loss. Ten participants partially completed the questionnaire and were removed from the analysis. This was because participants were assured that their data would not be included if they exited the questionnaire at any time. Therefore, the responses from 45 participants were included in the analysis.

Responses to the insulin restriction question were on a 5-point Likert scale, with the options ‘often’, ‘sometimes’, ‘rarely’ ‘not anymore’ and ‘never’. This was based on a similar approach used by Goebel-Fabri and colleagues [25], as social desirability pressures could influence participants to under-report insulin restriction. Therefore ‘rarely’ and ‘not anymore’ answers would still categorise participants as eligible for the study, only the answer ‘never’ would not. In the final sample, 38% stated they manipulated their insulin often, 33% not anymore, 27% sometimes and 2% rarely.

Forty-two participants identified as female, two identified as male and one chose not to say. There was no minimum age requirement for this study, as Diabulimia often affects mid-to-late teenagers as well as adults. Ages ranged from 15 to 58, with a mean age of 32.09 (*SD* = 11.07).

Participants were recruited in several ways. Posters were placed around the University of Central Lancashire campus and a link to the questionnaire was posted on social media. The study was shared online by Diabetics with Eating Disorders and Diabetes Daily. The link was also posted on a private Facebook group, ‘*Diabulimia Awareness*’.

### Materials

#### Eating disorder examination questionnaire (EDE-Q)

The EDE-Q [28] is a validated self-report alternative to the well-established Eating Disorder Examination (EDE), a clinician-administered interview [29]. The EDE-Q is a 28-item questionnaire, designed to measure the participant’s eating disorder psychopathology over the last 28 days. There are four subscales (restraint, eating concern, shape concern and weight concern) which combine to form the global score. A higher global score indicates greater eating disorder psychopathology. This is supported by research that has found the global score to be highly accurate in discriminating individuals with an eating disorder from those without [30].

#### Qualitative questions

Participants were first asked what age they were when they started to restrict insulin. Descriptive statistics were calculated for this. They were then asked 16 open-ended questions developed specifically for this study, focused on reasons for insulin restriction, triggering factors, medical experiences, and recovery. The responses were analysed using thematic analysis.

### Procedure

It was decided to conduct the research as an online survey rather than as interviews to maximise participation and ensure a mixed population of age and location.

Upon clicking the questionnaire link, potential participants were first briefed on the study and requested to confirm that they had read and fully understood the information provided. They were then asked if they consented to taking part. If consented, they were asked if they had T1DM and had ever restricted insulin to lose weight. Participants who answered no or never were disqualified from completing the questionnaire. Eligible participants gave their age and gender, before moving on to the EDE-Q and qualitative questions. Finally, participants were debriefed and thanked for their time.

Ethical approval was granted by the University of Central Lancashire Ethics Committee.

## Results

### Data screening

The normal distribution of the EDE-Q data was examined with analysis of the skewness and kurtosis values for each subscale and global scores. All values fell within the acceptable range of +/− 1.96, except the skewness for the shape concern subscale (− 3.60). Outliers were detected within the shape concern subscale, however as these values were within the normal range of scores for the scale they were not removed.

### EDE-Q analysis

The overall means and standard deviations for the subscale and global scores were calculated, represented in Table 1. These were compared to two general population samples [28, 31] and two eating disorder population samples [32, 33].

**Table 1:** Overall means and standard deviations for the EDE-Q compared to general population and clinical eating disorder samples

		General Population		Eating Disorder	
Scale	Present study	Fairburn and Beglin (1994)	Mond et al. (2006)	Brewin et al. (2014)	Rø, Reas and Stedal (2015)
Restraint	3.08 (1.76)	1.25 (1.32)	1.30 (1.40)	3.93 (1.65)	–
Eating Concern	3.47 (1.53)	0.62 (0.86)	0.76 (1.06)	3.85 (1.37)	–
Shape Concern	4.84 (1.16)	2.15 (1.60)	2.23 (1.65)	4.83 (1.23)	–
Weight Concern	4.44 (1.29)	1.59 (1.37)	1.79 (1.51)	4.49 (1.37)	–
Global Score	3.96 (1.21)	1.55 (1.21)	1.52 (1.25)	4.25 (1.20)	4.00 (1.32)

As shown in Table 1, the global score for the present study (3.96) is substantially higher than the global scores from general population samples (1.55 and 1.52), and more comparable to the global scores from eating disorder samples (4.25 and 4.00). Independent sample t-tests revealed that there was a significant difference between the present study’s mean global score and the mean of the general population global scores (t (1) = − 161.68, *p* = .004) and no significant difference between the present study’s global score and the mean of the eating disorder global scores (t (1) = 1.32, *p* = .413).

In addition, Rø, Reas and Stedal [32] suggest a global cut-off score of ≥ 2.50 when using the EDE-Q. In the present study’s sample, 38 (84%) scored above this cut-off. Therefore, it was assumed that the study was valid, as most of the sample would be considered to have clinical eating disorder psychopathology.

### Average age of insulin omission

The mean age of first restricting insulin was 19.31 (7.70), the minimum 12 and the maximum 45.

### Thematic analysis

The analysis was conducted by the corresponding author. Survey responses were read several times before a thorough and comprehensive coding procedure took place. Codes were collated into themes, which were then reviewed and defined. Due to limited theory in this area, an inductive approach at the semantic level was used in the development of themes [27].

#### Reasons for insulin restriction

When participants were asked why they restricted insulin, three main themes were identified: weight loss, a hate of diabetes and self-harm. Thirty-five out of the 45 participants (78%) stated that weight loss was the main reason they restricted their insulin,

*“Straight out of diagnosis I started to gain weight when I injected insulin, so I stopped”.*

It was discovered that they could eat whatever they wanted and still lose weight quickly and easily, which became an obsession,

*“Rather die than be fat”.*

*“I became obsessed with the number on the scale”.*

Eight participants (18%) described a hate of diabetes and wanting to regain control as their main reason for restricting insulin.

Any aspect of diabetes would be avoided as they were tired of it or did not want to be different from others,

*“Avoidance of thinking about being diabetic was the biggest reason I didn’t always take my insulin”.*

*“Escaping being diabetic”.*

Additionally, participants expressed feelings of having no control over their bodies, with the misuse of their insulin helping to regain that control,

*“It’s one thing that I thought controlled me and by not taking insulin I felt like I had the control back”.*

*“In control, like I had a little secret”.*

Two participants (4%) stated that the main reason they misused insulin was to self-harm,

*“Being high numbs me … It’s a way of self-harm, punishment if I have resentment towards myself”.*

The ten participants who stated that weight loss was not the main reason for their insulin restriction commented that it was a secondary reason.

#### Triggering factors

Participants reported a variety of factors that contributed to their misuse of insulin. The main themes were trauma, mental health comorbidities and feeling unsupported.

Participants described emotional, physical, and sexual abuse, parental separation, breakdowns in the family, and bullying,

*“My father left after I was diagnosed with type 1”.*

*“My step-father raped me and was sent to prison”.*

*“Bullying played a part in needing to lose weight and overall disgust at my reflection”.*

The most common comorbid mental health difficulties discussed were other eating disorders, depression, and stress. Participants often described having nobody to talk to, due to feelings of shame and a lack of understanding from others,

*“I feel that people may judge me”.*

*“I’m too ashamed to talk about it”.*

#### Medical experiences

There were three emerging themes to these questions: medical or psychiatric intervention, medical consequences, and negative experiences with professionals.

Thirty-three out of the 45 participants (73%) stated that they had received medical or psychiatric intervention because of their insulin misuse. The majority discussed how this was for treatment of DKA, with many becoming seriously ill on multiple occasions,

*“I nearly died last Friday due to DKA”.*

*“I’ve been in DKA more times than I can remember”.*

When asked about their experience with professionals, thirty-one participants (69%) reported this to be negative,

*“They just don’t understand, they think it’s as simple as ‘just take your insulin’”.*

*“Ignorance and misunderstanding. They just don’t get it, have never heard of it, or don’t understand how stuck you are”.*

Seven reported no experience and the remaining seven discussed a mixed or positive experience,

*“Physical support was fantastic but I believe more emotional support would have helped me accept my condition sooner …*”.

Overwhelmingly, 39 participants (87%) reported that they knew how serious the consequences of Diabulimia were, but found it too difficult to stop restricting insulin,

*“I knew exactly what I was doing, I knew I could probably only do it for a year or two before I died”.*

*“I am a doctor and know all the consequences and even suffer from many of them, neuropathy, nephropathy, but I can’t stop”.*

#### Recovery

When asked about recovery there were three main themes in responses: support, motivation, and awareness.

The support theme included the benefits of positive relationships, such as with family, children, and other sufferers online,

*“The diabulimia awareness site know exactly how I feel and give unconditional support”.*

*“I started a relationship with an extremely supportive partner who has helped me manage my diabetes”.*

The benefits of psychological support were also discussed,

*“Acceptance of my body through DBT”.*

*“There should be psychological support from diagnosis all the way through”.*

Participants described the benefits of having personal motivators such as university, as well as feeling motivated to minimise long-term health complications,

*“I’m doing my A Levels in a few months and it was having a huge impact on my work meaning I might not be able to go to uni … which has made me really want to change”.*

*“I’m still struggling but my feet hurt, my hair is falling out, my legs are not working like normal. I don’t want to be in a wheelchair and bald at 40 so I’m trying to get better”.*

Most participants discussed a need for greater awareness and understanding of Diabulimia, both for professionals and people with diabetes,

*“EVERY nurse, psychologist, dietician … should know what the signs are and be trained to help guide the person to appropriate treatment”.*

*“I think that more awareness of Diabulimia is needed and that some sort of training or information sessions would be of great benefit to type 1 diabetics and their families …*”.

All participants felt that Diabulimia was viewed negatively. They described stigma and a lack of understanding, even within diabetes and eating disorder communities,

*“Absolutely stigmatised! Our bodies don’t get as small as women with anorexia and we weight recover quickly so clinicians are quick to dismiss us even though we’ll be back in emergency with DKA in a few days”.*

## Discussion

The aim of this study was to explore Diabulimia from the perspective of those with lived experience. It was hoped that identified themes would help to guide the focus of future research. Additionally, it was aimed to raise awareness and understanding of Diabulimia at a time when it is rightfully gaining attention within medical and psychiatric communities.

### Clinical implications

In support of past research, weight loss and a fear of weight gain were the main reasons participants restricted insulin [10, 22, 25, 26]. It was also identified that the difficulties of living with diabetes, such as feeling a lack of control, were core factors in the emergence of Diabulimia. This adds to previous research which found that struggling to live with a long-term condition puts people with diabetes at risk of developing eating disorders [13]. Also, many participants had experienced traumatic events such as abuse, family breakdown, and bullying. This supports research that has linked trauma exposure to eating disorders [34, 35]. The findings of the present study highlight the importance of psychological intervention for Diabulimia, specifically intervention that targets trauma and beliefs about weight and appearance. Additionally, interventions should focus on helping individuals to cope with a long-term condition.

Most participants had required medical or psychiatric intervention for Diabulimia, often due to serious or life-threatening complications. An overwhelming 87% of individuals were aware of the serious consequences of Diabulimia but chose to continue restricting insulin. This supports findings from one small sample of insulin restrictors [22] but contradicts the findings of another [10]. As the current study has a much larger sample, the findings are likely to be more representative. The NICE guidelines state that sufferers should be offered education on the consequences of insulin restriction [5]. However, people with diabetes are often ‘expert patients’ and do not need to be taught the importance of physical health monitoring and the consequences of poor diabetes management [6]. The findings from this study suggest that effective psychological treatment should follow any diabetes education. Current clinical practice guidelines for eating disorders recommend psychological treatment such as Cognitive Behavioural Therapy and Family Based Therapy [5]. To deliver these effectively, clinicians need to understand the specific issues associated with insulin omission.

The importance of positive relationships was common in responses, supporting previous research [8, 22] and suggesting that a focus on relational aspects would be important in treatment. Individuals strongly felt that improved support from professionals is needed, with only 15% reporting positive or mixed experiences. This highlights the need for a shift in the way professionals view Diabulimia. More education is crucial, particularly for those who support people with eating disorders and/or diabetes. To effectively support the medical and psychological needs of people with Diabulimia, it is essential for collaboration between physical and mental health professionals [6, 13].

The age of commencing insulin restriction ranged from 12 to 45. This supports research that has found eating disorders to occur in midlife as well as in adolescence and young adulthood [36, 37]. Therefore, professionals should be aware that there is a risk for Diabulimia in older as well as younger people with diabetes.

### Limitations

The survey only obtained two male responses, and therefore the results may only be applicable to women experiencing Diabulimia. Women are more likely to be diagnosed with eating disorders and therefore eating disorder research tends to focus on women [38]. Despite this, research has found that a quarter of eating disorder cases are men [39]. Males with eating disorders often underreport symptoms and a lower proportion participate in research [39]. Future studies should aim to investigate potential gender differences in people with Diabulimia.

In addition, the study would have benefitted from including additional demographic data. The inclusion of international participants will have enhanced the generalisability of the findings, but future research should seek to uncover any differences between countries.

Although the online questionnaire allowed for varied data and easy accessibility, semi-structured interviews would obtain more detailed and thorough information. Future studies could build on the present findings by conducting interviews with people who restrict insulin.

Finally, due to the reliance on self-report data, it is possible that participants had a wide range of insulin restriction severity and frequency. The results may also be impacted by selection bias, as people with more severe insulin misuse may have been more likely to participate in the study.

## Conclusions

Psychological support should be at the forefront of treatment for people with Diabulimia. To deliver effective psychological treatment, clinicians must understand the physical and psychological issues associated with insulin restriction. The current study presented the lived experience perspective and identified themes that may be helpful for clinicians to consider and explore. These include concerns about weight or appearance, difficulties adjusting to diabetes, past trauma, and the importance of supportive relationships. It is hoped that these results will help to increase awareness and understanding of Diabulimia. Future research should aim to build on these results and explore the identified themes further.

## Data Availability

The datasets generated and analysed during the current study are not publicly available due to participant confidentiality. Anonymous parts of the material can be available by contacting the corresponding author.

## Abbreviations

T1DM: Type 1 diabetes mellitus
EDE-Q: Eating Disorder Examination Questionnaire
NICE: National Institute for Health and Care Excellence
DEB: Disturbed eating behaviours
BMI: Body mass index
DKA: Diabetic ketoacidosis
